# Variability in frost occurrence under climate change and consequent risk of damage to trees of western Quebec, Canada

**DOI:** 10.1038/s41598-022-11105-y

**Published:** 2022-05-04

**Authors:** Benjamin Marquis, Yves Bergeron, Daniel Houle, Martin Leduc, Sergio Rossi

**Affiliations:** 1 Département des Sciences Fondamentales, Université du Québec à Chicoutimi, 555 boulevard de l’Université, Chicoutimi, QC G7H 2B1 Canada; 2 Institut de recherche sur les forêts, Université du Québec en Abitibi-Témiscamingue, 445 boulevard de l’Université, Rouyn-Noranda, QC J9X 5E4 Canada; 3 Département des Sciences Biologiques, Université du Québec à Montréal, 141 avenue du Président-Kennedy, Montréal, QC H2X 1Y4 Canada; 4 Division Science et Technologie, Environnement et Changement Climatique Canada, Montréal, QC Canada; 5 Consortium sur la climatologie régionale et l’adaptation aux changements climatiques (OURANOS), 550 Sherbrooke Ouest, Montréal, QC H3A 1B9 Canada; 6 Present Address: Natural Resources Canada, Canadian Forest Service, Great Lakes Forestry Center, 1219 Queen Street East, Sault Ste. Marie, ON P6A 2E5 Canada

**Keywords:** Physiology, Plant sciences, Climate sciences, Ecology, Environmental sciences, Natural hazards, Ecology, Boreal ecology, Climate-change ecology, Ecophysiology, Forest ecology

## Abstract

Climate change affects timings, frequency, and intensity of frost events in northern ecosystems. However, our understanding of the impacts that frost will have on growth and survival of plants is still limited. When projecting the occurrence of frost, the internal variability and the different underlying physical formulations are two major sources of uncertainty of climate models. We use 50 climate simulations produced by a single-initial large climate ensemble and five climate simulations produced by different pairs of global and regional climate models based on the concentration pathway (RCP 8.5) over a latitudinal transect covering the temperate and boreal ecosystems of western Quebec, Canada, during 1955–2099 to provide a first-order estimate of the relative importance of these two sources of uncertainty on the occurrence of frost, i.e. when air temperature is < 0 °C, and their potential damage to trees. The variation in the date of the last spring frost was larger by 21 days (from 46 to 25 days) for the 50 climate simulations compared to the 5 different pairs of climate models. When considering these two sources of uncertainty in an eco-physiological model simulating the timings of budbreak for trees of northern environment, results show that 20% of climate simulations expect that trees will be exposed to frost even in 2090. Thus, frost damage to trees remains likely under global warming.

## Introduction

Climate is a major force of adaptation and evolution determining fitness of individuals (survival and reproduction) and species range limits^1–3^. In extratropical ecosystems, plants exhibit a dormant phase during which they increase their cold tolerance by extracting water to the extracellular space, concentrating sugar inside the cells and producing proteins that protect enzymes and the cellular membrane from freezing and dehydration^4^. These processes take place at the end of summer to prevent damage from early frost events, and gradually subside in spring with the decreased risk of frost^2,3^. As a result, there is no threshold for differentiating a frost event causing damage to trees. A damage is expected when the frost event exceeds the frost hardiness of plant tissues, conditions that are most likely in spring and fall, when trees have lower resistance to low temperatures^5^. Once damaged by frost, plant development can be importantly hindered^6,7^. For instance, frost damage to the newly formed leaves reduces the photosynthetic capacity of trees and limits growth^8^. If the apical meristem is damaged, the growth stagnation problem is aggravated, and trees affected by frost can lose the competition for space to trees or shrub species that were unaffected by the frost^6,7^. Therefore, the processes of adaptation and acclimation of plants in cold climates must match the timings of weather events such as daily frosts, rather than the average climate conditions such as annual or monthly temperatures^9,10^.

By increasing the frequency and intensity of extreme weather events, global warming can modify the environment at a rate that exceeds species adaptation and acclimation abilities^11,12^. If the rate of warming in spring that triggers the reactivation of the meristems of trees is faster than the rate at which frost days decrease, trees could reactivate their meristems under greater risk of freezing. Determining how the ongoing warming will affect frost occurrence becomes critical to adequately predict growth and survival of forest ecosystems under changing climatic conditions. However, the challenges related to predicting when, where, at what frequency and intensity daily frosts could occur limited the use of this climate variable in phenological models and for determining the plant functional types in dynamic global vegetation models^13–15^. As a result, the potential impact of early and late frosts on plant phenology is still poorly understood.

Recent advances in the development of large climate ensembles can help to better quantify the occurrence of future daily weather from a same climate model and representative concentration pathway [RCP] scenario^16,17^. By sampling several climate trajectories within the range of internal (natural) climate variability, they can be used to estimate for a given model and scenario, the probability of occurrence of frost events for a specific day in the year for both present and future climates^18–21^. Therefore, these single model initial conditions large climate ensemble can help to overcome the challenges in simulating the future occurrence of frosts.

Even when performing inter-climate model comparison in climate change risk analysis, studies still neglected the variation in daily weather induced by the internal variability in a climate model^22,23^. For instance, for the same climate model, location and CO_2_ concentration scenario, several climate simulations with slight differences in their initial conditions can produce different daily outputs^19–21^. Because daily weather events rarely occur, a greater variation in their occurrence among simulations is expected compared to the variation in averages, such as mean annual temperature. To date, most analyses regarding the importance of the internal variability of a climate model on climate projections have considered conventional aggregated temperature and precipitation metrics such as mean annual temperature, and snowpack, as well as some weather events such as droughts, heavy rainfall, and storms^24–26^. However, the role of the internal variability of a climate model on the occurrence of daily frosts is still lacking, despite its importance for climate change risk assessment in cold regions.

**Figure 1:**
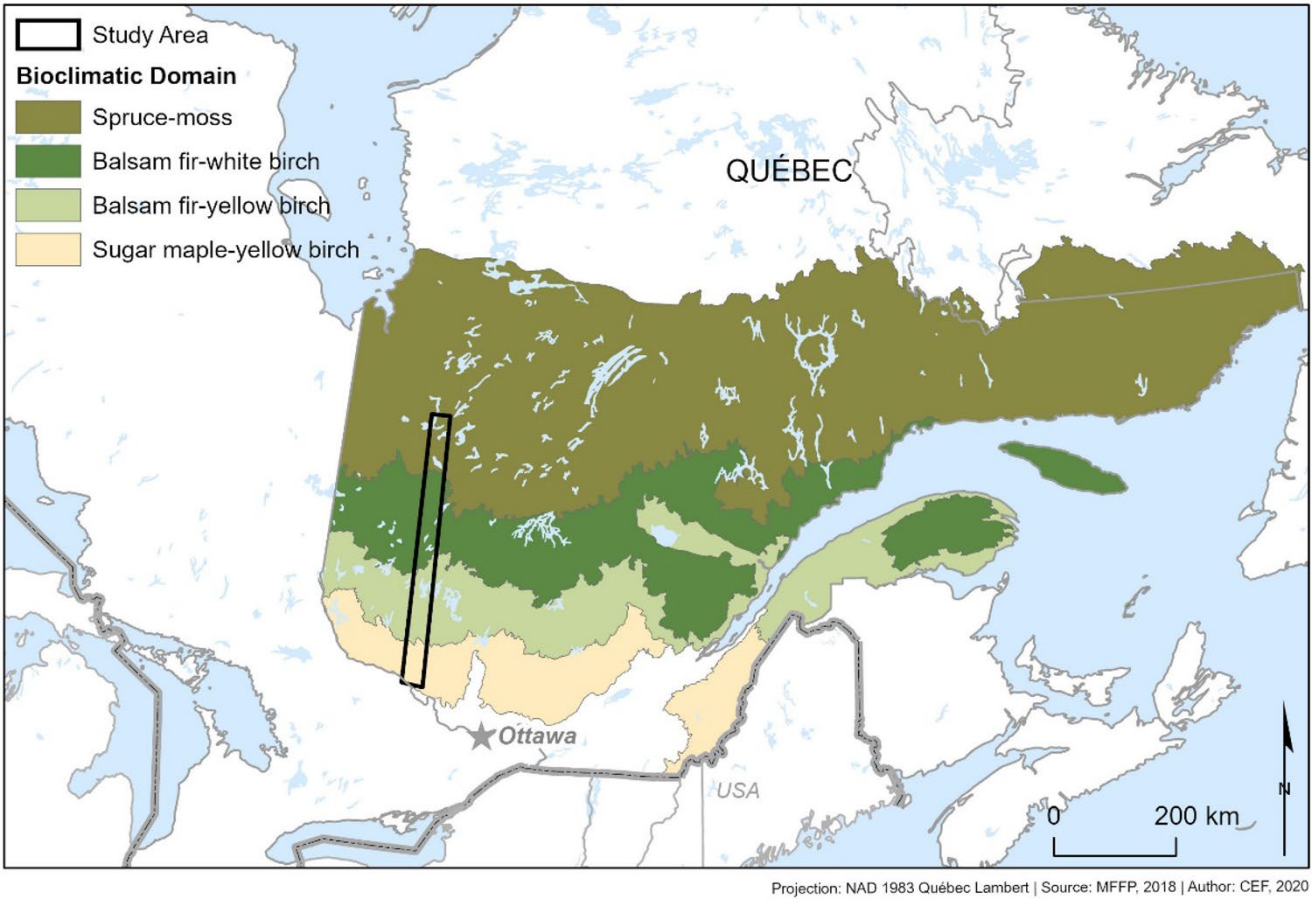
Map of eastern Canada showing the latitudinal transect and different bioclimatic domains covered by our study area. The software ESRI’s ArcGIS Pro 2.8 was used to produce the map.

We designed our study to challenge the cutting-edge problem related to simulating the occurrence of daily frost events under climate change and consequences for climate change risk assessment. We aimed to distinguish the effect of two sources of uncertainty in climate models: the internal variability of a climate model and the underlying formulation of different climate models on future projections of daily frost occurrence. We also aimed to assess the impact of the variation in climate on budbreak simulations and exposure to late-frost of temperate and boreal tree species over a latitudinal transect covering the boreal-temperate forest of Quebec during 1955–2100 (Fig. 1). We assessed the uncertainty in expected frost occurrence caused by the internal variability of a climate model by analyzing the spread in expected frost occurrence between 50 climate simulations produced by a single-initial large climate ensemble based on a single pair of regional and global climate models hereafter referred to as the intra-model variability. The uncertainty caused by the underlying formulation of different climate models was assessed by analyzing the spread in expected frost occurrence produced by five climate simulations using different combinations of global and regional climate models hereafter referred to as inter-model variability. Since increasing the number of climate simulations improve the detection of rare events such as late frosts, we expected that the use of a single-initial-large-ensemble produced larger variations in the timings of frost occurrence than the five climate simulations generated by different pairs of global and regional climate models.

## Results

### Internal variability and projected climate trends

Along the transect, the mean annual temperature between 1955 and 1984 varied from 4.4 °C in the South (< 46° N) to 0.5 °C in the North (> 48.5° N) (inter- and intra-model variability is shown in panels A,E of Fig. S1 respectively). During this period, the intra-climate model variation in mean annual temperature ranged from 2.2 to 7.3 °C in the South and from − 2.5 to 3.8 °C in the North (panels A,E in Fig. S1). For 2070–2099, models projected an increase in the mean annual temperature up to 11.0 and 8.0 °C in the South and North (inter- and intra-model variability is shown in panels A,E in Fig. S1). During 2070–2099, the intra-climate model variability is expected to range from 8.2 to 13.8 °C in the South and from 4.3 to 10.7 °C in the North (panels A,E in Fig. S1).

The growing degree-days (GDD) between 1955 and 1984 varied from 2810 in the South to 2153 in the North (panels B,F in Fig. S1). During this period, the intra-climate model variations in GDD ranged from 2361 to 3370 in the South and from 1507 to 2672 in the North. In 2070–2099, the intra-climate model variability projected GDD to range from 3684 to 5283 in the South and from 2827 to 4694 in the North with respective averages of 4483 and 3744 (panels B,F in Fig. S1).

**Figure 2:**
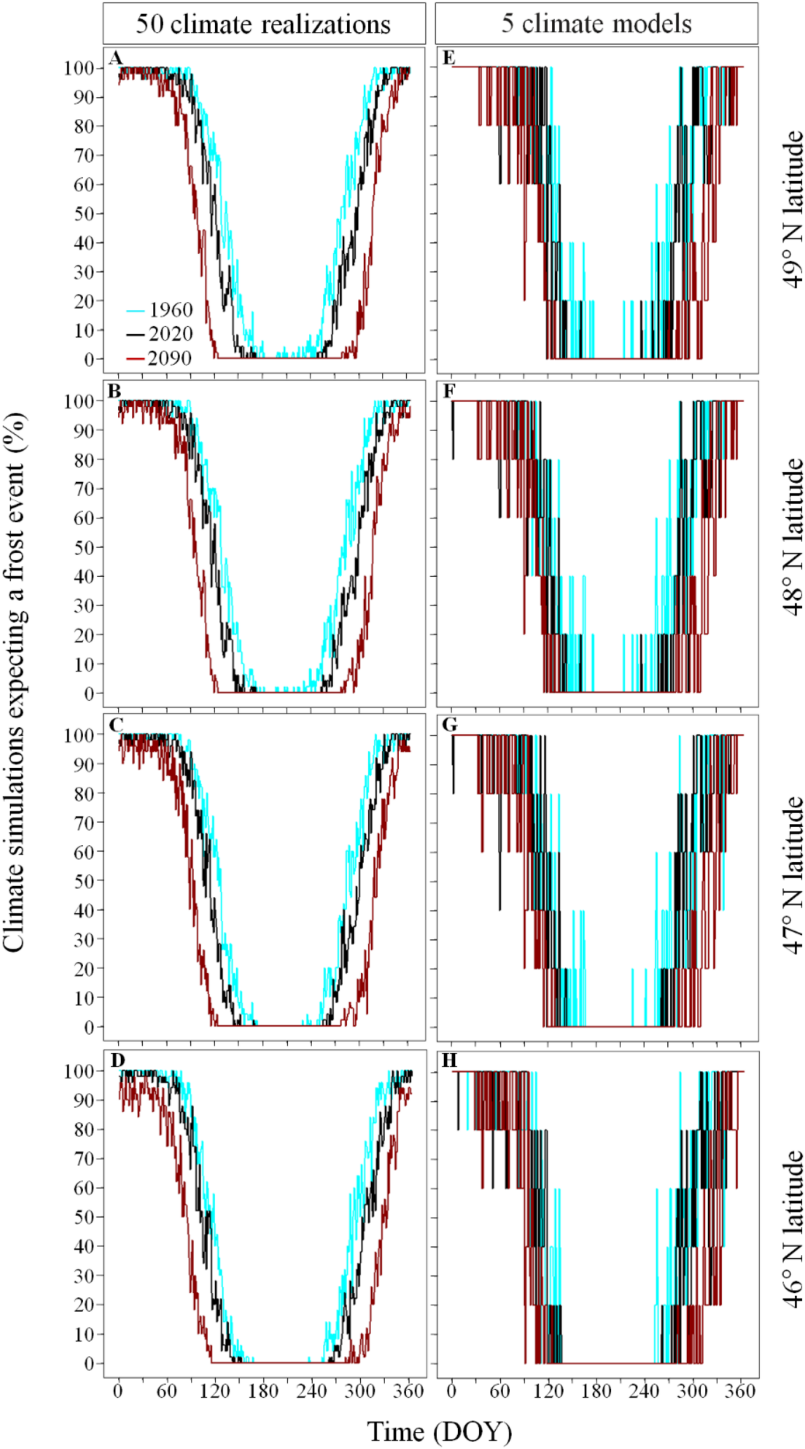
Percentage of climate simulations predicting a frost event per day of year along the latitudinal gradient for three specific years (1960, 2020, 2090). The columns show the results of the intra- and inter-climate model analyses and the rows identify specific grid cells along the latitudinal gradient (46.08, − 77.53; 47.07, − 77.33; 48.21, − 77.40; 49.01, − 77.44 for the intra-climate model analysis: and 46.09, − 77.28; 47.05, − 77.59; 48.17, − 77.50; 49.03, − 77.17 for the inter-climate model). We used the ggplot2 library developed by Wickham^27^ to produce the figure.

**Figure 3:**
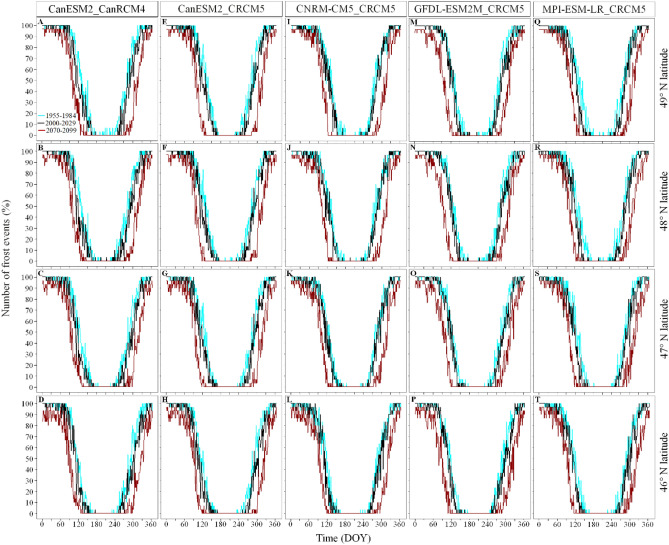
Percentage of frost events for each day of the year over three specific periods (1955–1985, 2000–2030, 2070–2099) along the latitudinal gradient per climate model used in the multi-climate model ensemble. The columns show the results of each climate model and the rows identify specific latitudes along the latitudinal gradient (46.09, − 77.28; 47.05, − 77.59; 48.17, − 77.50; 49.03, − 77.17). We used the ggplot2 library developed by Wickham^27^ to produce the figure.

The frost-free period in 1955–1984 varied by 35 days along the latitude, lasting 124 days in the South (from DOY 142 [panels C,G in Fig. S1] to 265 [panels D,H in Fig. S1]), and 89 days in the North (from DOY 161 to 249). During this period, the intra-climate model variation in beginning and ending dates of the frost-free period were of 67 and 70 days respectively in the South, and 79 and 88 days in the North (panels G,H in Fig. S1). For 2070–2099, the frost-free period is expected to last 200 days in the South (from DOY 104 to 304), and 185 days in the North (from DOY 115 to 300). During this period, the intra-climate model variation in beginning and ending dates of the frost-free period were of 71 and 63 days respectively in the South, and 54 and 63 days in the North (panels D,H in Fig. S1). As a result, the intra-climate simulations produced by the pair of regional and global climate model CanESM2_CRCM5 expects that the frost-free period advanced by 38 days in the South and 46 days in the North during spring compared to a delay of 39 and 51 days in the South and in the North respectively during fall (Fig. 2). This equitable contribution of the warming in spring and fall to the increased length of the frost-free period is also observed for each of the pairs of global and regional climate models used in the multi-model ensemble (Fig. 3). On average, the five climate models project that future (2070–2099) warming will advance spring by 37 days and will delay fall by 38 days (Fig. 3).

### Spatial and temporal trends of frost occurrence

**Figure 4:**
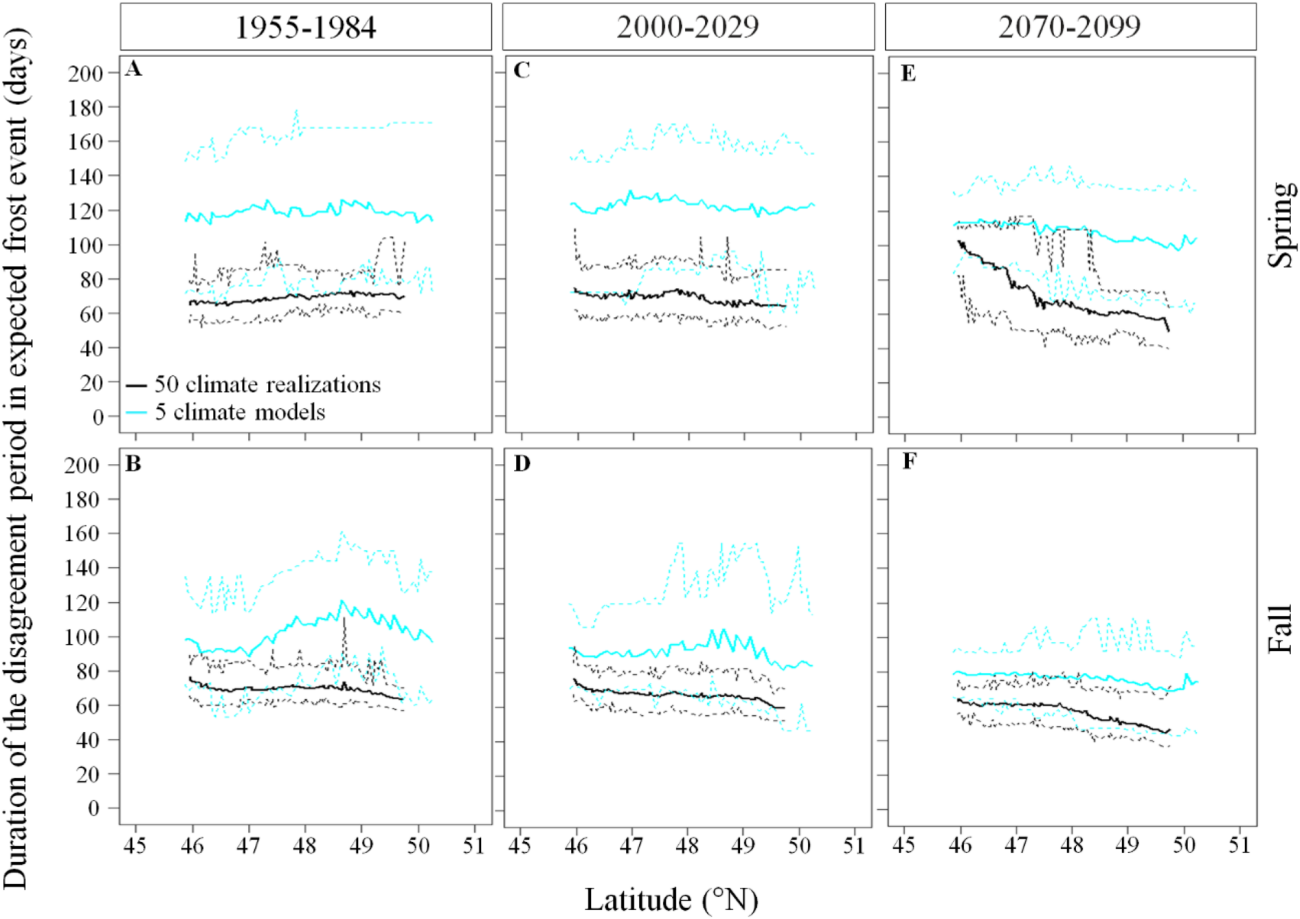
Inter- and intra-climate model variability in the duration of the disagreement period in expected frost occurrence during spring and fall across latitudes and simulation time. Solid lines represent the mean, and dashed lines the maximum and minimum durations of the disagreement period in expected frost occurrence per season and 30-year periods respectively. The disagreement period in expected frost occurrence refers to the period when the percentage of climate simulations expecting the occurrence of frost are < 90% and > 10% for the intra-climate model (internal variability) analysis and are < 80 and > 20% for the inter-climate model (difference between model formulations) analysis since it is during this period that climate simulations diverge the most in their expected frost occurrence. We used the ggplot2 library developed by Wickham^27^ to produce the figure.

During 1955–2099, climate simulations agreed on their expected frost occurrence during winter (> 90% of climate simulations expect a daily frost event) and during summer (< 10% of climate simulations expect a daily frost event) but disagreed during spring and fall (Fig. 2). Based on the inter-climate model analysis, the duration of these disagreements lasted, on average and over the whole study area, 117 ± 21 and 90 ± 18 days, respectively. Based on the intra-climate model analysis, the duration of the disagreement in expected frost occurrence during spring and fall were 47 and 24 days shorter than the inter-climate model variability, lasting, on average and over the whole study area, 70 ± 12 and 66 ± 8 days. In all cases, the duration of these two disagreements were similar across latitudes and years (Fig. 4).

**Figure 5:**
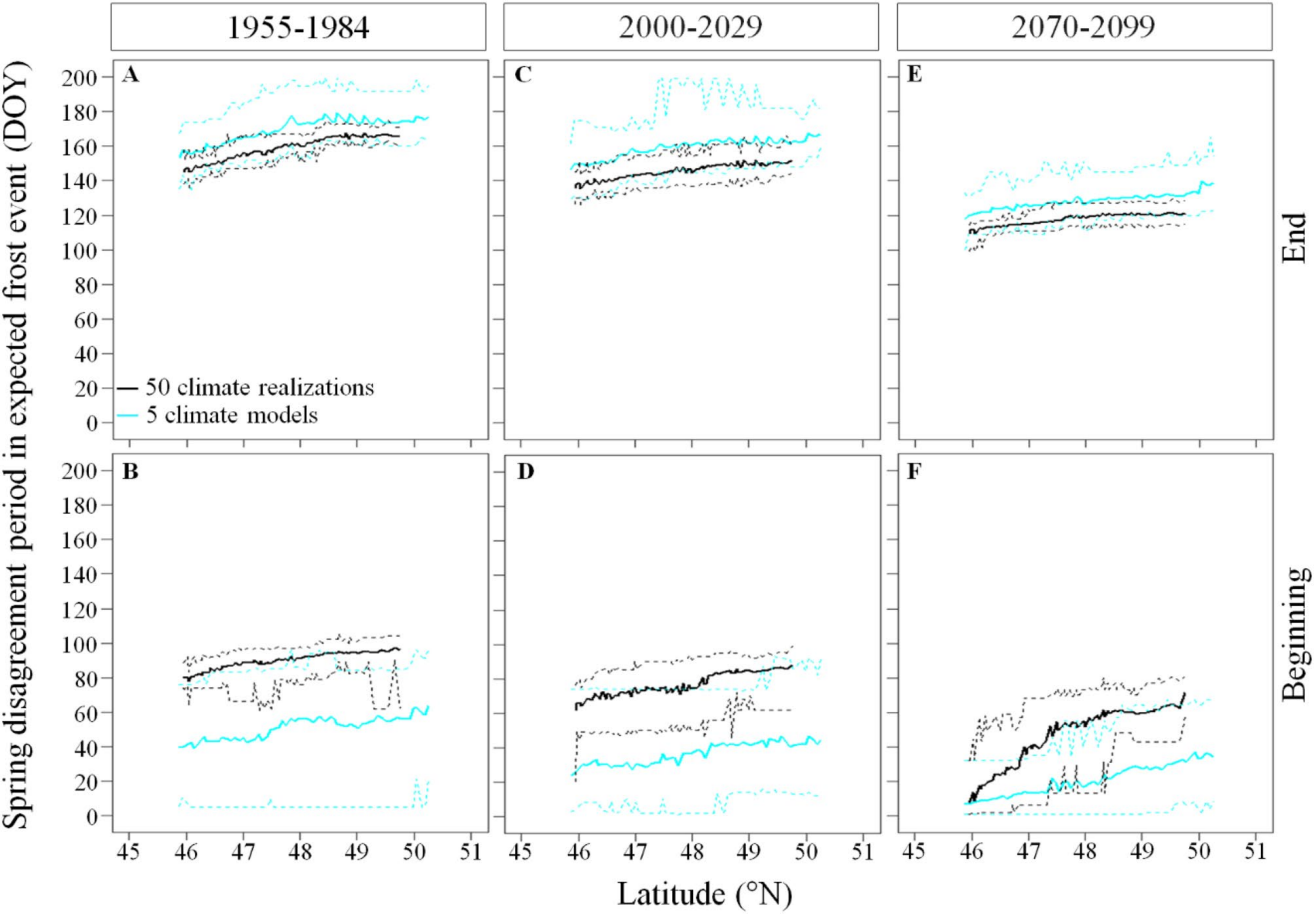
Inter- and intra-climate model variability in the day of year of the beginning and end of the disagreement period in expected frost occurrence during spring across latitudes and 30-year periods. Solid lines represent the mean, and dashed lines the maximum and minimum DOY determining the beginning and end of the disagreement period in expected frost occurrence across latitudes. The disagreement period in expected frost occurrence refers to the period when the percentage of climate simulations expecting the occurrence of frost are < 90% and > 10% for the intra-climate model (internal variability) analysis and are < 80 and > 20% for the inter-climate model (difference between model formulations) analysis since it is during this period that climate simulations diverge the most in their expected frost occurrence. We used the ggplot2 library developed by Wickham^27^ to produce the figure.

**Figure 6:**
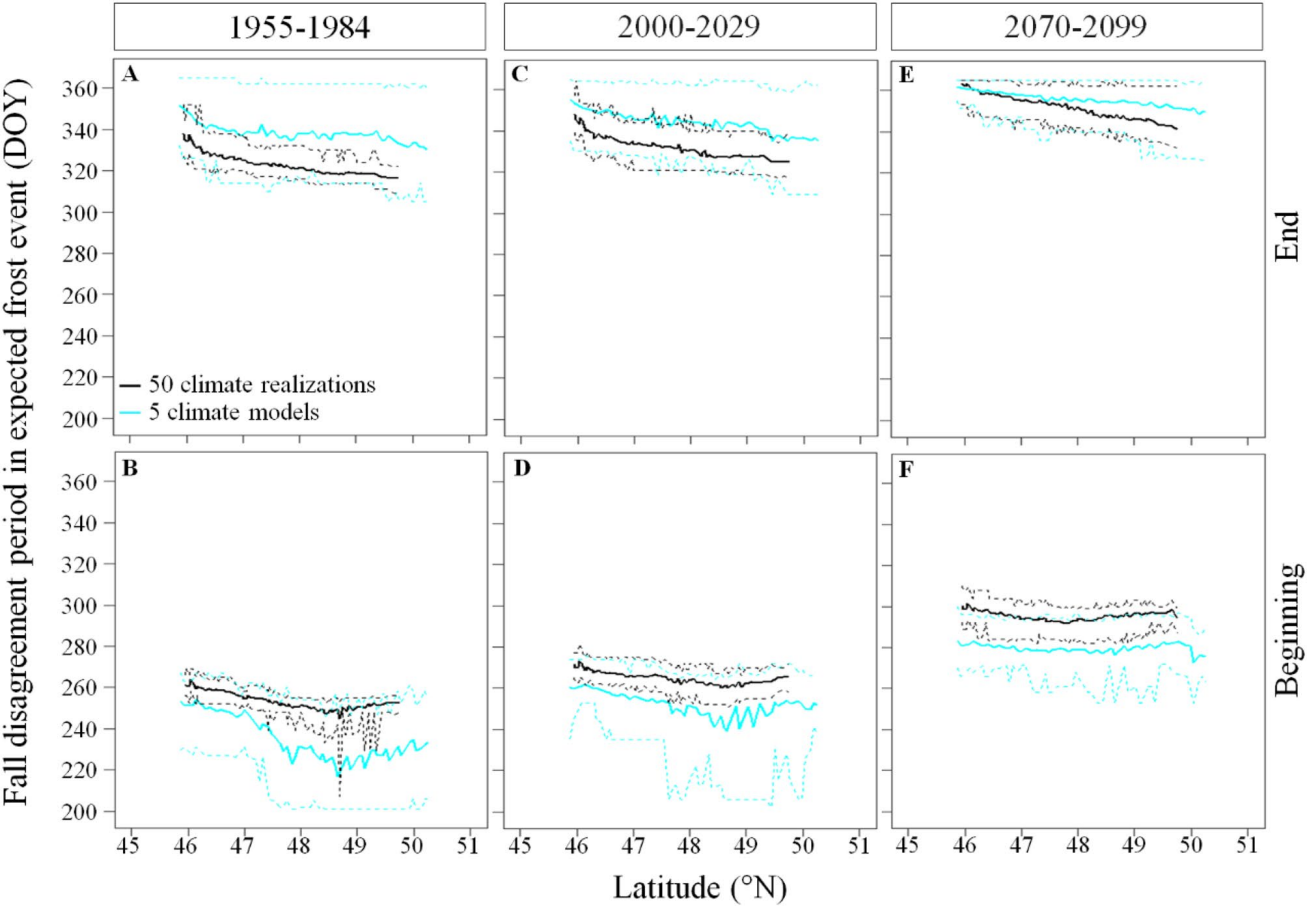
Inter and intra climate model variability in the day of year of the beginning and end of the disagreement period in expected frost occurrence during fall across latitudes and 30-year periods. The solid lines represent the means, and the dashed lines the maximum and minimum DOY determining the beginning and end of the disagreement period in expected frost occurrence across latitudes. The disagreement period in expected frost occurrence observed between climate models and within a climate model refers to the period when < 90% and > 10% of climate simulations expect a frost event since it represents the period during which frosts may or not occur, hence models disagree in their expected frost occurrence. We used the ggplot2 library developed by Wickham^27^ to produce the figure.

The intra-model analysis showed important spatial and temporal trends for the beginning and ending dates of the disagreement during spring and fall (Table S1). During 1955–1984 we show that the start of the disagreement in expected spring frost events occurred later at increasing latitude (panels A,B of Fig. 5), from DOY 80 (ranging from DOY 73 to DOY 89) in the South to DOY 95 (ranging from DOY 62 to DOY 105) in the North. By 2070–2099, the start of the spring disagreement period is expected to occur earlier by 72 days in the South and by 34 days in the North. During 2070–2099, the end of the spring disagreement period is expected to advance to DOY 121 in the South and to DOY 110 in the North. During 1955–1984, the fall disagreement period started 10 days later in the South compared to in the North, from DOY 261 (ranging from DOY 256 to DOY 269) to DOY 251 (ranging from DOY 207 to DOY 256) and also ended 19 days later in the South, from DOY 337 (ranging from DOY 326 to DOY 352) in the South to DOY 318 (ranging from DOY 309 to DOY 330) in the North (panels A,B of Fig. 6). By 2070–2099, the start of the fall disagreement period is expected to occur 39 and 45 days later in the South and in the North respectively (panel F of Fig. 6). However, the end of the disagreement period in fall is expected to occur 26 and 28 days later (panel E of Fig. 6). Hence, the disagreement period in fall is expected to be shorter in the future since it lasted 76 and 67 days from South to North during 1955–1984 and are expected to last 63 and 50 days by 2070–2099 (Fig. 6).

The inter-model comparison showed that during 1955–1984 the start of the disagreement in expected spring frost events occurred later at increasing latitude, from DOY 40 in the South to DOY 56 in the North (panels A,B of Fig. 5). During this period the variability in the start of the disagreement period in spring was of 70 days in the South, ranging from DOY 5 to DOY 71 and 91 days in the North, ranging from DOY 5 to DOY 96. However, the variability in the ending dates of the spring disagreement period was shorter than the variability in the starting dates of the spring disagreement period, lasting 39 days in both the South and the North. Even if the end of the spring disagreement period had similar duration, it showed temporal variation since it ranged from DOY 135 to DOY 174 in the South and from DOY 160 to DOY 199 in the North. The start and the end of the fall disagreement period also showed spatial and temporal trends as well as large variabilities (Fig. 6). During 1955–1984, the fall disagreement period started on DOY 252 in the South and on DOY 228 the North. The variability in the start of the disagreement period lasted 37 days in the South (from DOY 230 to DOY 267) and lasted 60 days in the North (from DOY 201 to DOY 261). During 2070–2099 the start of the fall disagreement period is expected to start 30 days later in the South (ranging from DOY 266 to DOY 300) and 52 days later in the North (from DOY 253 to DOY 297). However, the end of the fall disagreement period showed smaller variability and reduced spatial and temporal trends compared to its starting date. For instance, the end of the fall disagreement period is expected to occur 10 and 17 days later during 2070–2099 compared to 1955–1984 in the South (from DOY 351 to DOY 361) and in the North (from DOY 335 to DOY 352) respectively.

### Variability in the projected budbreak period

**Figure 7:**
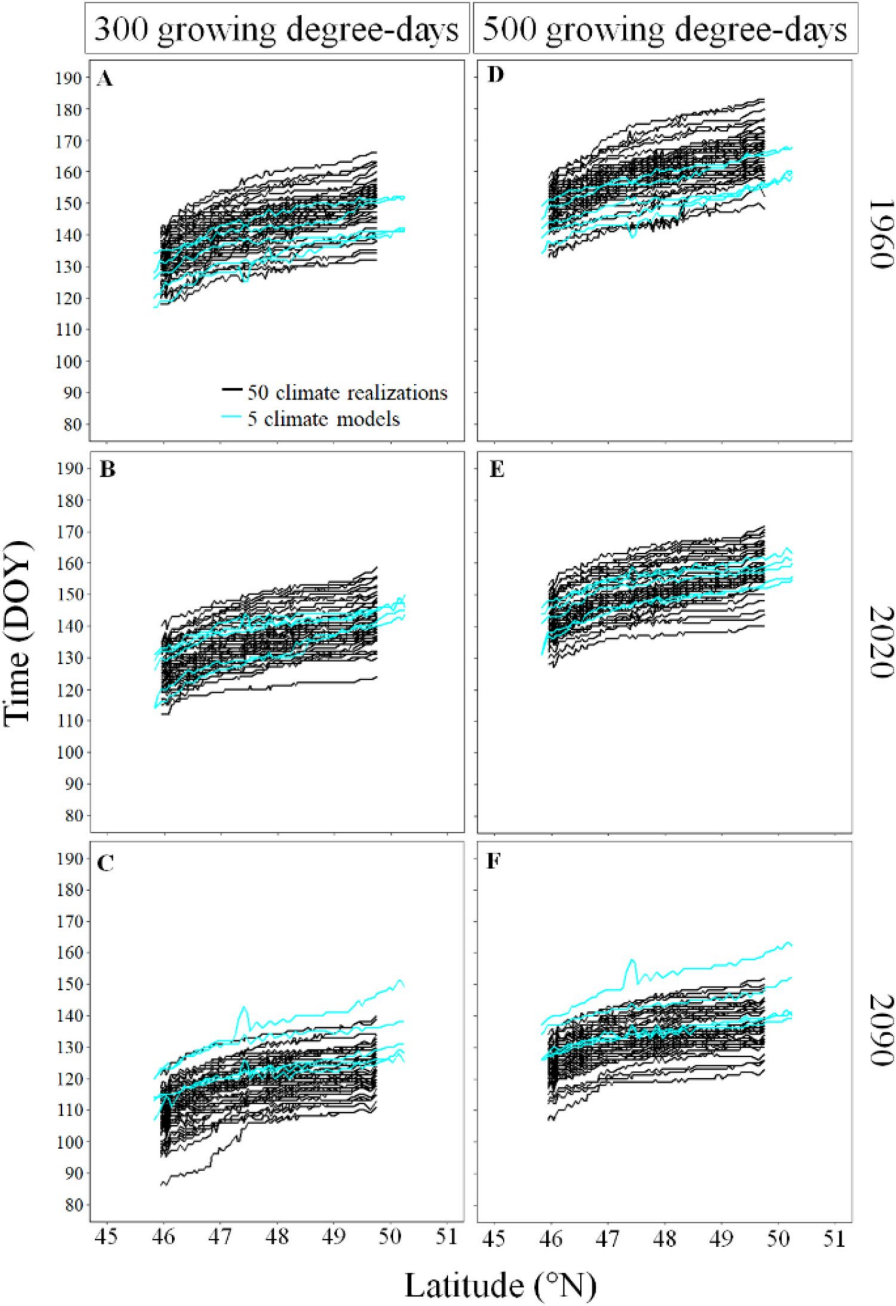
Inter- and intra-climate model variability in the day of year of the probable budbreak period along the latitudinal gradient for three specific years (1960, 2020, 2090). The columns show the growing degree-day threshold used to represent early and late leaf-out species (300 and 500 GDD) and the rows show the specific years on which budbreak is expected to occur. We used the ggplot2 library developed by Wickham^27^ to produce the figure.

The duration of the estimated budbreak period, i.e. the difference between earliest and latest day of the year when climate simulations reached 300 (panels A–C of Fig. 7) and 500 GDD (panels D–F of Fig. 7), was similar across the latitudinal gradient, and was on average 29 and 27 days for the intra-climate model analysis and 15 and 14 for the inter-climate model analysis respectively (Fig. 7). Thus, according to our data, the internal variability of the global (CanESM2) and regional (CRCM5) climate chain led to larger spread in the possible budbreak period than the 5 pairs of global and regional climate chains used for the inter climate model analysis (see Table S2 for the summary statistics). Similar trends also appeared at the daily scale (see Figs. S2, S3 for the inter- and intra-climate model variation in GDD and minimum air temperature over a year).

### Variability in the risk of late frost exposure to trees

**Figure 8:**
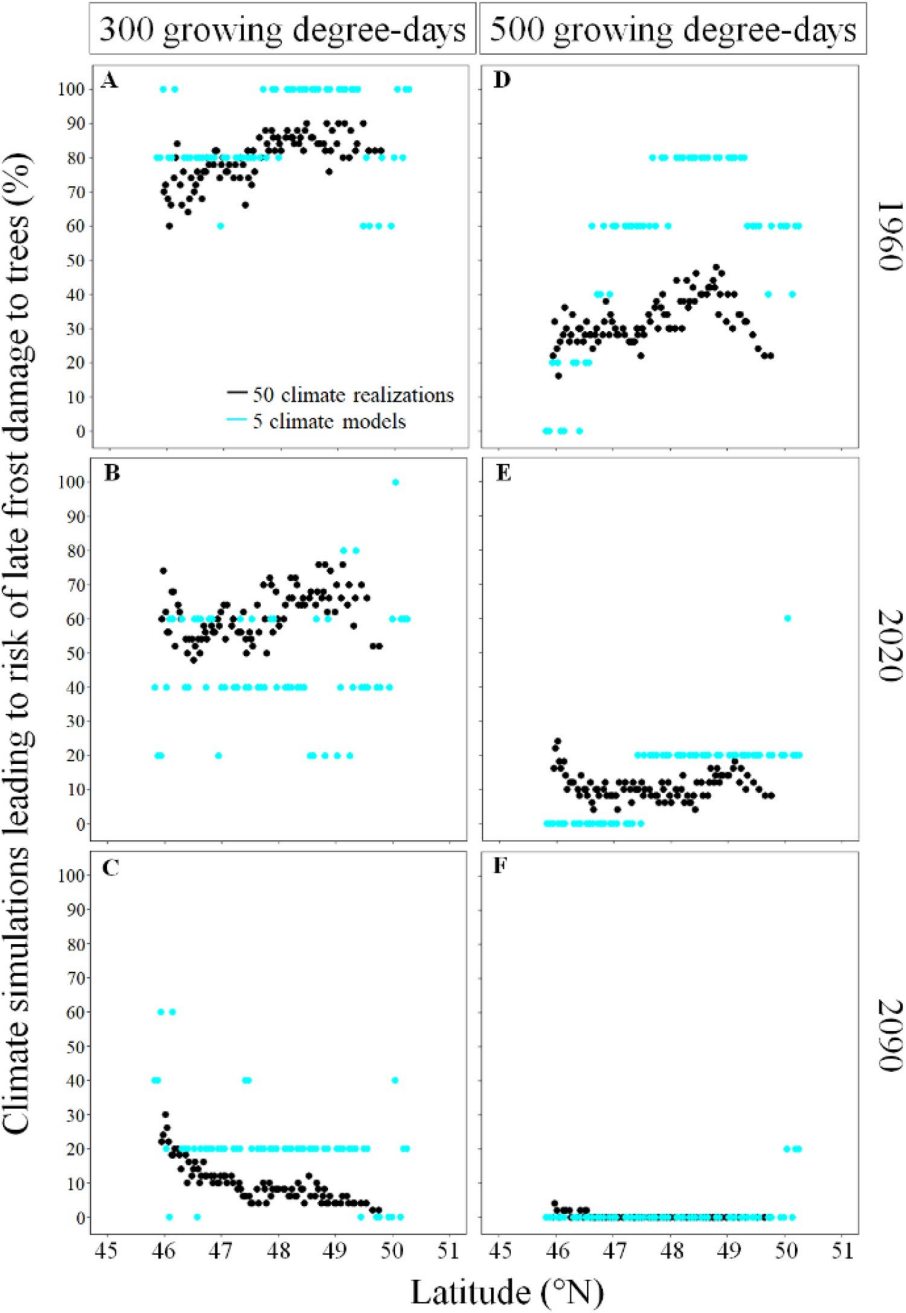
Inter- and intra-climate model variability in projected risk of frost exposure to early and late leaf-out species along the latitudinal gradient for three specific years (1960, 2020, 2090). The columns show the growing degree-day threshold used to represent early and late leaf-out species (300 and 500 GDD) and the rows show the specific years. We used the ggplot2 library developed by Wickham^27^ to produce the figure.

In the past (1955–1984), the risk of late frosts increased from 74 to 83% with increasing latitude (panels A–D of Fig. 8). This trend reverses in the future (2070–2099), decreasing the risk of late frosts from 23 to 12% from South to North (panels C–F of Fig. 8). All over the transect, early leaf-out species (theoretical value of 300 GDD) were more subjected to risk of late frosts compared to late leaf-out species (theoretical value of 500 GDD). For instance, we show that 0 to 96% of climate simulations predicted risk of late frosts to early leaf-out species (panels A–C of Fig. 8) compared to 0–56% for late leaf-out species (panels D,F of Fig. 8), with averages of 48 and 14%, respectively. During 1955–1984, the average risk of late frost to late leaf-out species also increased South to North from 23 to 35% whereas, during 2070–2099, it was close to 0, ranging from 0 to 8%.

The inter-model analysis showed that between 1955–1984 and 2070–2099, the risk of late frosts decreased on average from 79 to 19% and from 30 to 3 for early leaf-out and late leaf-out species respectively (Fig. 8). However, during 1955–1984, the risk of late frost damage to early leaf-out species increased from 71 to 81% from South to North whereas, during 2070–2099 the spatial trend reversed, decreasing from 25 to 19% from South to North. During 1955–1984, the spread of the expected risk in late frost to early leaf-out species ranged from 0 to 100 in the South and from 40 to 100 in the North but during 2070–2099 it ranged from 0 to 80 all along the latitudinal gradient. For late leaf-out species, the spread of the expected risk in frost damage during 1955–1984 ranged from 0 to 60% in the South and from 0 to 100% in the North whereas, during 2070–2099, it ranged from 0 to 40% all along the latitudinal gradient. Thus, according to our data providing a first-order estimate of the variability in frost projections, both the inter- and intra-model analyses in risk of late frost to tree species revealed similar spatial and temporal trends (panel C of Fig. 8). However, the spread of the expected risk in late frost to early leaf-out species was larger for the inter-model analysis compared to the intra-model analysis, ranging from 0 to 100% and 54 to 96% respectively during 1955–1984 and from 0–80 to 0–36% respectively during 2070–2099 (Table S3). Including more than five chains of global and regional climate models might have revealed a larger spread in expected risk in late frost to early leaf-out species. Thus, the comparison between the intra- and inter-model analyses remain to be validated using larger sets of climate models.

## Discussion

### Inter- and intra-climate model variability of frost occurrence

The wider variability in the duration of the disagreement period in late frost observed in the inter-climate model during 1955–1984 was mainly caused by advances in the starting dates, since one of the five chains of global and regional climate models predicted an air temperature above 0 °C on January 1st compared with DOY 61 predicted by the intra-climate model variation. Compared to the CanESM2_CRCM5 intra-climate model variability, the wider inter-climate model variability in duration of the disagreement period for early frost expectations was mostly observed at the northern most latitudes and was caused by an earlier starting date toward summer. Since trees of northern environment enter dormancy sooner than trees from southern environment, trees are resistant to freeze–thaw events, and even early frosts are less likely to damage trees. If each pair of regional and global climate model has an internal variability generating a variability as wide as that expected by the pair of climate model we used, there is evidence that the variability in expected frost occurrence in spring and fall are largely underestimated. Given that our study provides a first-order estimate of the variability in projected frost, a thorough assessment of the variability in frost projection using many pairs of global and regional climate model is necessary.

### Variability in budbreak predictions

Climate variables such as GDD, which sum the temperatures above a specific threshold varied greatly between simulations since each small variation in day-to-day weather accumulates over the year, leading to different days of year when a specific GDD threshold is reached. This intra-climate model variability in the CanESM2_CRCM5 chain remained constant along the latitudinal gradient, as also observed by Leduc et al.^19^ who showed that the intra-climate model variability in monthly mean temperature mostly increased in northern latitudes above our study transect. The intra-climate model variability in GDD was also wider than the inter-climate model variability for the past, current, and future periods. Our results only partly support the hypothesis made by Deser et al.^18^ that the internal climate variability plays a major role in predicting climate trajectory during the early years of a climate simulation since our results from the intra-climate model analysis did not decreases in importance for long-term climate predictions. Hence, the responses of climate models to the increase in CO_2_ emission did not add more variability to growing degree-days than the internal variability of a climate model even if the internal variability tended to remain constant across years. However, recall that it was the case for the duration of the disagreement period in spring frost occurrence since the inter-model analysis projected this period to last 117 days whereas the internal variability of the pair of climate model CanESM2_CRCM5 projected that it lasted 70 days. This increased variability in future temperature predictions is named model response uncertainty, and results from the difference in parameterization and structure between climate models and represents a constraint in reliably modeling the climate dynamics^23^. Based on our results, we conclude that the interpretation on the accuracy of long-term climate projections is highly dependent on the climate variable under study and generalizations on the accuracy of long-term climate projections should not be attempted. We are aware that we have only used one initial condition large climate ensemble to analyze the internal variability of climate models and few global climate models that were downscaled by only two regional climate models to analyze the inter-climate model variability. Therefore, our results only provide a first estimate of the total variability existing if all global and regional climate models were paired. Even in case of an underestimation of the inter-model variabilities in frost projections, the large internal variability in the chain of global (CanESM2) and regional (CRCM5) climate model suggests that the weather forecasts, such as the frosts, can be strongly influenced by the internal variability of climate models. Hence, some uncertainties seem to be intrinsic and irreducible components of a climate model. Our results suggest the need to proceed with a coordinated intercomparison effort extending our experimental framework to the use of several dynamical and statistical downscaling models as climate input to multiple ecological models to properly asses the risk of climate change to ecosystems.

### Risk of frost damage to trees under climate change

The frost-damage hypothesis, which suggests that climate warming will induce an earlier budbreak thus increasing the exposure of vulnerable tree tissues to spring frost, is under debate due to contrasting results^28–32^. This discrepancy in the risk of frost damage prediction is often attributed to the fact that ecophysiological models predicting budbreak timing and seasonal changes in frost hardiness are rarely calibrated with the results of observations or experiments at both inter- and intra-species level. Consequently, they could fail to provide accurate budbreak predictions in synchrony with frost predictions within a species range, with important impacts on the expected risk of frost damage^32,33^. However, our results suggest that this discrepancy in expected risk of frost damage to trees could be attributed to the climate simulation that was used. For instance, we showed that the internal variability of the model chain CanESM2_CRCM5 could vary annually by 46 days in the date of the last predicted frost event between climate simulations. Similarly, based on a thermal-process based model, the expected date of budbreak for a given year could also vary by 29 days, which leads to 0–60% of climate simulations predicting growing season frosts even in 2090. Since climate variables are used as input values in process-based models predicting bud phenology, growth and leaf senescence and only a few climate simulations are used, the wide variation in daily weather expected by climate models is not captured by these models of leaf phenology. As a result, the consequence of the ongoing warming on vegetation properties and ecosystem functions is likely underestimated. If frost importantly impacts the current vegetation, in the long-term, we might observe a change in the vegetation toward more frost resistant species. This change in vegetation might impact the local thermal land–atmosphere and create feedback that in turn alter the occurrence of frost. Since frost damage has greater consequences on the fitness of seedlings compared to mature trees^7^, this potential vegetation-thermal feedback might only occur in the long term. However, because dynamic global vegetation models neglect the effect of frost on plants, this feedback and its potential effect to the carbon and nutrient cycles can hardly be evaluated.

Our theoretical budbreak simulation synchronized with late frosts showed that early leaf-out species will be more exposed to late frost compared to late leaf-out species. Moreover, in the future (2070–2099), late frost occurrence is expected to decrease and extend to the temperate regions rather than to the boreal forest. Importantly, all evidence suggests that advancement in spring phenology is worthy of more concern than the potential exposure to early frosts in fall. First, the rate at which leaf senescence is delayed is expected to decrease because bud set and growth cessation are primarily under photoperiod and genetic control^34–38^, therefore, the warmer temperatures will unlikely extend the growing season later in the fall. Second, spring and fall phenology were reported to be synchronized^39^. As a result, an earlier spring reactivation due to the ongoing warming should correspond to an earlier fall dormancy^40^, which would also decrease the risk of damage to trees by early frost. Third, the advance in budbreak timing already increased the risk of late frost damage to trees whereas increased damage from early frost still needs to be shown^28–30^. Consequently, in the future, damage to temperate and boreal trees by early frost is, according to current knowledge, less likely than damage from late frosts.

Since the future variability in frost projections for the temperate-boreal forest ecotone remains wide, selecting tree species with high resistance to cold and strategies to late frost avoidance such as late leaf-out will be important functional traits to consider for optimizing the productivity of the planted trees^41^. Therefore, forest management in temperate and boreal forests should not only aim to limit the negative impact of droughts on tree productivity and mortality^42–44^ but should also aim to limit late frost damage to trees. First, new plantations should be established on high terrain and trees should be planted on mounds since cold air tends to accumulate at the bottom of slopes and in topographic depressions^45,46^. Second, assisted migration which consists of the human-based movement of species or populations possessing adaptations that should allow better growth than local species or populations under the future climate may help to preserve forest productivity in a context of climate change^47,48^. However, when climate is too stressful, the increased growth of non-local compared to local species or populations is reduced^49^. Therefore, when moving southern species or seed sources towards the north, cold adaptation, and the timing of leaf phenology (budburst and bud set) should be of primary concern since these traits determine which seed sources can support cold during winter and avoid late frosts. However, traits most often measured in provenance trials and common gardens focus on growth such as tree height and diameter^50^. While short-term studies found that moving southern species or seed sources northward did increase tree productivity^51^, a long-term study found that this positive effect was of short duration (< 15 years), which might not significantly increase wood production over the rotation time, which lasts around 60–90 years in boreal ecosystems^52^. Therefore, including cold adaptation and frost avoidance strategies should help to identify the best species or seed sources to plant under the future climate. Ecotypes with late-leaf out may increase long-term efficiency of assisted migration since they should avoid damage from late frosts^41^.

It is important to note that we used two theoretical GDD thresholds (300 and 500) but due to adaptation to local climate, the threshold triggering bud opening in northern tree populations may be lower compared to southern populations^53^. Moreover, we did not consider the intensity of the frost events and having used 0 °C as threshold value determining potential frost damage to trees, we likely overestimated the risk. Still, sustainable forest management should consider the potential negative impacts of late frosts on trees, even under climate warming. Importantly, the risk of frost exposure we calculated over the latitudinal transect is theoretical and aimed to show the wide variation in expected risk of frost exposure that is currently unaccounted for in dynamic global vegetation models^14,15^.

By providing a first estimation of the uncertainties around the predicted occurrence of frost, we highlighted new insightful knowledge on the importance of two sources of uncertainties in climate models (1) the internal variability of climate models and (2) the different physical formulations of climate models when simulating the response of trees of northern ecosystems to climate change. The five different combinations of GCM and RCM used in our study remain a fraction of all potential combinations. Thus, deeper investigations regarding the effect of the physical formulations of climate model on the expected frost occurrence will be required. Moreover, it would be interesting to compare results of frost occurrence obtained from the dynamic downscaling of global climate model to regional climate model to a statistical downscaling.

While there was a general agreement in the level of warming projected for spring and fall between climate models, not all pairs of regional and global climate models project the same warming. Nonetheless, we showed that the warming in spring and fall could mismatch the cycle of dormancy and growth from the seasonal variations in weather that plants are adapted to. Determining the ability of trees to deal with these changing conditions becomes a priority for research in plant ecology. By determining the proportion of climate simulations predicting the risk of late frost we could provide important practical information for developing adaptive strategies mitigating the negative impact of climate change to northern ecosystems. In addition to the direct damage to plants, leafless trees impact surface albedo. Therefore, the plant functional types used in dynamic vegetation models to group plant species bearing similar eco-physiological characteristics such as leaf longevity, photosynthetic pathway, deciduous or evergreen and herbaceous or woody^14,15^ should integrate leaf phenology and the impact of frost on trees. This may improve the accurateness of Earth system models (ESMs) in predicting the response of ecosystems to climate change. Importantly, this new method assessing inter- and intra-climate model variability in expected frost occurrence could also apply to all extreme weather events and their consequence on ecosystem functioning. Evaluations of the risk of late frost to temperate and boreal tree species based on multiple climate simulations should become a common procedure when assessing climate change risk assessment.

## Materials and methods

### Study area

The study area consists in a latitudinal transect covering 5° along the parallels (46 to 50° N) and 2° along the meridians (− 78 to − 76° W) in Quebec, Canada (Fig. 1). This area represents the range between deciduous temperate and boreal forest and encompasses four bioclimatic domains, i.e. zones characterized by similar vegetation and climate^54^. This change in vegetation represents a change in the mean annual temperature from 5 °C in the south (Climate Normals 1981–2010, Environment Canada, Sheenboro weather station) to 0 °C in the north (Climate Normals 1981–2010, Environment Canada, Joutel weather station). Since air temperature often oscillates around 0 °C, this area allows a suitable assessment of the level of importance of the inter- and intra-climate model variation in expected frost occurrence.

### Single-model initial-condition large ensemble

To analyze the impact of the internal climate variability on the expected occurrence of frost events, which is considered as days for which the minimum air temperature is below 0 °C, we used the bias-corrected daily minimum air temperature simulated by 50 different climate simulations produced by pair of regional (CRCM5) and global (CanESM2) models using different boundary condition forcing to assess the impact of initial conditions. These simulations were run on a grid with 12 × 12 km of spatial resolution (88 grid cells covering our study area) over the period from 1955 to 2099 and following the representative concentration pathway (RCP) 8.5. The air temperature data was corrected using an additive quantile mapping based on the 99 empirical percentiles of the daily time series, using the 1971–2000 period in NRCan 10-km Canada Daily as the reference dataset^55^. The correction function was calculated using detrended data for each DOY and grid cell separately, with a 15-day moving window^56,57^. This set of 50 simulations is one of the two model ensembles developed in Canada, and its greater resolution (12 *vs* 50 km grid resolution) directed our choice^19,26^. Because of the logistical constraints related to running these multiple simulations at such a high resolution, such as computation power, only one RCP scenario was run. Still, this extreme scenario could project the widest variability in frost risk since the warmer temperatures in spring already advanced budbreak timing and increased the exposure of frost sensitive tree tissues to late frosts^28–31^. Even if the use of downscaling with regional climate model is under debate, its additional value is clear when the objective focusses on weather events or over complex topography^58^, which is the case in our study. Indeed, frost events consist in rare local daily events that are strongly connected to the regional topography, since cold air accumulates at the bottom of slopes^59^. In addition, the biases in climate projections produced by different chains of global and regional climate models are not additive and downscaling regional climate models can reduce the biases in climate projections compared to only using global climate models^60^. Specifically, the internal variability of the CanESM2-CRCM5 ensemble, the ensemble used in our study, was able to reproduce the inter-annual variability of past air temperature and precipitation trends obtained from the E-OBS dataset over Western Europe^61^. Therefore, the dynamic downscaling of a global climate model with a regional climate model seems clearly justified.

### Multi-model ensemble

We quantified the variability in frost occurrence by using the bias-corrected daily minimum air temperature produced by a set of five pairs of global and regional climate models: (1) CanESM2_CanRCM4, (2) CanESM2_CRCM5, (3) CNRM-CM5_CRCM5, (4) GFDL-ESM2M_CRCM5, and (5) MPI-ESM-LR_CRCM5 (the global climate model is shown first separated by an underscore from the regional climate model). The abovementioned post-processing procedure was used to obtain bias-corrected air temperature data^55,56^. The climate simulations were produced at a spatial scale of 22 km × 22 km (58 grid cells covering our study area) over the same period (1955–2099) and for the same representative concentration pathway (RCP) 8.5 as was used for the single large climate model ensemble so that we could compare the climate simulations produced by both inter and intra-climate models. Only one pair of regional and climate model (CanESM2_CanRCM4) had a different RCM and grid than the other four pairs. During the application, we merged the closest grid cells together so that all five models matched the same grids, which facilitates the comparison of the simulations. Since some models consider the leap year and others did not, we removed every February 29 to compare models with the same calendar of 365 days.

### Climate model uncertainty and internal variability in frost occurrence

When climate simulations agree on their expected frost occurrence, all of them predict the occurrence (like in winter) or absence (like in summer) of a frost event. When simulations diverged in their expectations of frost occurrence, we considered that these climate simulations disagree from one another. We determined the level of agreement in expected frost occurrence between climate simulations by calculating the percentage expecting a frost event per day, year and grid cell. We considered that climate simulations agree when > 90% predicted the same event (occurrence or absence of frost), which generally occurs during winter and summer. We used the 90% threshold values instead of the commonly used threshold (95%) because each climate simulation contributes 2% (1/50) of the total, it was thus impossible to reach odd thresholds with an increment of 2. The 90% threshold is also commonly used in studies measuring beginning and ending dates of tree growth^62^. By using percentage, we could compare the level of variation in frost forecast by both inter- and intra-climate model analyses even with a different number of climate simulations (5 vs 50). We determined the period of disagreement in expected frost occurrence between climate simulations by subtracting the last DOY when > 10% forecasted a frost event in late spring to the first DOY when < 90% forecasted a frost event in early spring. To determine the period of disagreement in expected fall frost occurrence between climate simulations, we subtracted the last DOY when < 90% forecasted a frost event in late fall to the first DOY when > 10% forecasted a frost event in early fall. For analyzing the variation in frost occurrence expected by the five different pairs of regional and global models, the thresholds 10–90% were replaced with thresholds 20–80% since they represent the time when one simulation is different from the four others. The determination of the DOY when climate simulations agreed and disagreed was conducted in each grid cell so that this timing of agreement and disagreement between climate simulations could be compared along the latitudinal gradient (see “Variability in the expected frost occurrence” section).

### Variability in the risk of late frosts after budbreak

We used a thermal process-based model predicting the timing of budburst based on two theoretical values of GDD (300 and 500). According to the literature, these thresholds represent a general and representative range for local species with early [sugar maple (*Acer saccharum* Marshall), balsam poplar (*Populus balsamifera* L.), white spruce (*Picea glauca* Moench Voss), and white birch (*Betula papyrifera* Marsh.)^63–65^] and late [red oak (*Quercus rubra* L.), and black spruce (*Picea mariana* Mill. B.S.P.)^66^] leaf out. Such a simple model often outperforms other more complex models in predicting leaf-out dates^67^. We determined the inter- and intra-climate model variability in DOY when 300 and 500 GDD were reached by counting the number of days between the climate simulation leading to the earliest and latest DOY when 300 and 500 GDD were reached, which represents a probable period for budbreak. We determined the number of climate simulations expecting a frost event (minimum air temperature < 0 °C prior to DOY 200) once 300 or 500 GDD had been reached. In this study, we assumed that any late frost event occurring during budbreak or leafing could damage the new tissues^28^. According to Man et al.^68^, trees species of eastern Canada require at least 400 h of chilling unit, which were reached before DOY 365 in 99% of the climate simulations. Thus, we could confirm that chilling requirements were completed before the accumulation of the thermal units (Fig. S4). As a result, chilling played a marginal role in our predictions of budbreak.

### Variability in the expected frost occurrence

We analyzed the spatial and temporal trends in the duration, beginning and end of the disagreement period in expected frost occurrence using linear regressions. The number of days or the DOY when the disagreement period in expected frost occurrence began and ended were used as response variable and latitude, year, type of model (inter *vs* intra), and their interactions were used as factors. All analyses were performed in the R software for statistical computing (version 4.0.3)^69^.

### Variability in the risk of late frosts after budbreak

We used the same procedure for analyzing the spatial and temporal variability in budbreak and late frost exposure as we used analyzing frost predictions. We used a linear regression model with the day of year of the simulated budbreak timing or the percentage of simulations expecting a frost event after budbreak as response variable and latitude, year, type of model, and their interactions were used as factors.

## Supplementary Information

Supplementary Information.

## Data Availability

Since the climate data used in this study were generated by climate models and consist in extremely large files, the data are only available upon valid request.
