# Supplementary material for: Variability in frost occurrence under climate change and consequent risk of damage to trees of western Quebec, Canada
Source: Sci Rep. 2022 May 4;12:7220. doi: 10.1038/s41598-022-11105-y (PMC9068889; doi:10.1038/s41598-022-11105-y)
Supplement: Supplementary file 1 — Supplementary Information. [file 41598_2022_11105_MOESM1_ESM.docx]

**Table S1. Spatial and temporal trends in the duration, beginning, and ending day of the year when expected frost occurrence disagrees among climate simulations.** Boldface coefficients are statistically significant (n = 21170; P-value <0.05).

|  | **Duration** | | **Beginning** | | **End** | |
| --- | --- | --- | --- | --- | --- | --- |
|  | Spring  (adj. R^2^ = 0.72) | Fall  (adj. R^2^ = 0.64) | Spring  (adj. R^2^ = 0.76) | Fall  (adj. R^2^ = 0.85) | Spring  (adj. R^2^ = 0.88) | Fall  (adj. R^2^ = 0.70) |
| Intercept | **-7456.00 (205.40)** | **-3494.00 (144.60)** | **6118.00 (188.50)** | **3208.00 (103.10)** | **-1338.00 (90.94)** | **-285.60 (107.30)** |
| Latitude | **156.30 (4.31)** | **78.49 (3.03)** | **-109.70 (3.95)** | **-76.80 (2.16)** | **46.63 (1.91)** | 1.69 **(**2.25**)** |
| Year | **3.81 (0.10)** | **1.82 (0.07)** | **-3.18 (0.09)** | **-1.40 (0.05)** | **0.63 (0.04)** | **0.42 (0.05)** |
| Multi-model | **132.80 (13.33)** | **164.60 (9.38)** | **-126.60 (12.23)** | **-12.69 (6.69)** | **6.20 (5.90)** | **151.90 (6.96)** |
| Latitude × Multi-model | **2.05 (0.18)** | **2.50 (0.13)** | **-2.62 (0.16)** | **-0.61 (0.09)** | **-0.57 (0.08)** | **1.89 (0.09)** |
| Latitude × Year | **-0.08 (2.12 × 10^-3^)** | **-0.04 (1.49 × 10^-3^)** | **0.06 (1.95 × 10^-3^)** | **0.04 (1.07 × 10^-3^)** | **-0.02 (9.40 × 10^-4^)** | **-3.23 × 10^-3^ (1.11 × 10^-3^)** |
| Multi-model × Year | **-0.09 (5.06 × 10^-3^)** | **-0.13 (3.56 × 10^-3^)** | **0.11 (4.64 × 10^-3^)** | **0.01 (2.54 × 10^-3^)** | **0.02 (2.24 × 10^-3^)** | **-0.11 (2.64 × 10^-3^)** |

**Table S2. Spatial and temporal trends in day of the year required for budbreak.** Results for early and late leaf-out species (300 and 500 GDD) are shown in separate columns. All coefficients are statistically significant (n = 680050; P-value <0.0001).

| **Variable** | **300 growing degree-days**  **(adjusted R^2^ = 0.67)** | **500 growing degree-days**  **(adjusted R^2^ = 0.73)** |
| --- | --- | --- |
|  | Coefficients (std error) | Coefficients (std error) |
| Intercept: intra model | -149.60 (17.43) | -385.60 (16.17) |
| Latitude | 14.62 (0.37) | 20.55 (0.34) |
| Year | 4.87 ×10^-2^ (8.60 ×10^-3^) | 0.17 (7.97×10^-3^) |
| Multi-model:inter model | -69.14 (2.05) | -77.27 (1.90) |
| Latitude × Multi-model | 0.12 (0.03) | 0.14 (0.02) |
| Latitude × Year | -5.32 ×10^-3^ (1.80 ×10^-4^) | -8.23 ×10^-3^ (1.67 ×10^-4^) |
| Multi-model × Year | 0.03 (8.03 ×10^-4^) | 0.04 (7.45 ×10^-4^) |

**Table S3**. **Spatial and temporal trends in the percentage of climate simulations expecting frost damage to buds.** Results for early and late leaf-out species (300 and 500 GDD) are shown in separate columns. Asterisks show the significance level (*** for < 0.001, n= 21170).

| **Variable** | **300 growing degree-days**  (adjusted *R*^2^ = 0.73) | **500 growing degree-days**  (adjusted *R*^2^ = 0.50) |
| --- | --- | --- |
|  | Coefficients (std error) | Coefficients (std error) |
| Intercept | -1960.00 (200.00) *** | -2500.00 (156.00) *** |
| Latitude | 67.30 (4.19) *** | 64.60 (3.28) *** |
| Year | 0.99 (0.10) *** | 1.23 (0.08) *** |
| Multi-model | -218.00 (13.00) *** | -86.60 (10.20) *** |
| Latitude × Multi-model | 1.43 (0.17) *** | 0.04 (0.14) |
| Latitude × Year | -0.03 (2.07× 10^-3^) *** | -0.03 (1.62 ×10^-3^) *** |
| Multi-model × Year | 0.08 (4.95× 10^-3^) *** | 0.04 (3.88 ×10^-3^) *** |


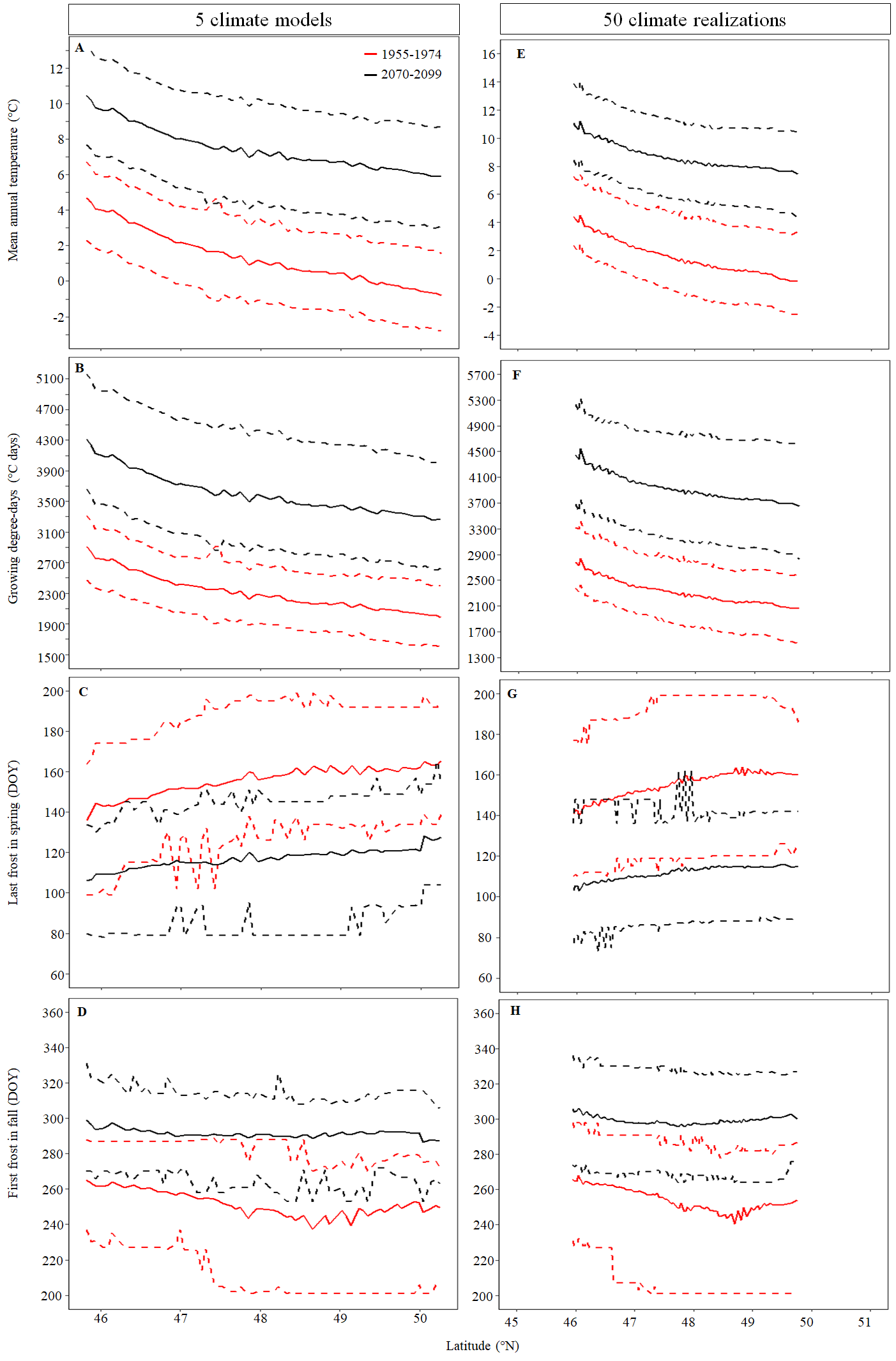


**Fig. S1. Inter and intra-model variability in climate metrics (mean annual temperature panels A and E, growing degree-days panels B and F, last frost in spring, panels C and G, and first frost in fall, panels D and H) along the latitude.** Solid lines represent the mean, and dashed lines the maximum and minimum values. Colors differentiate periods under analyses.


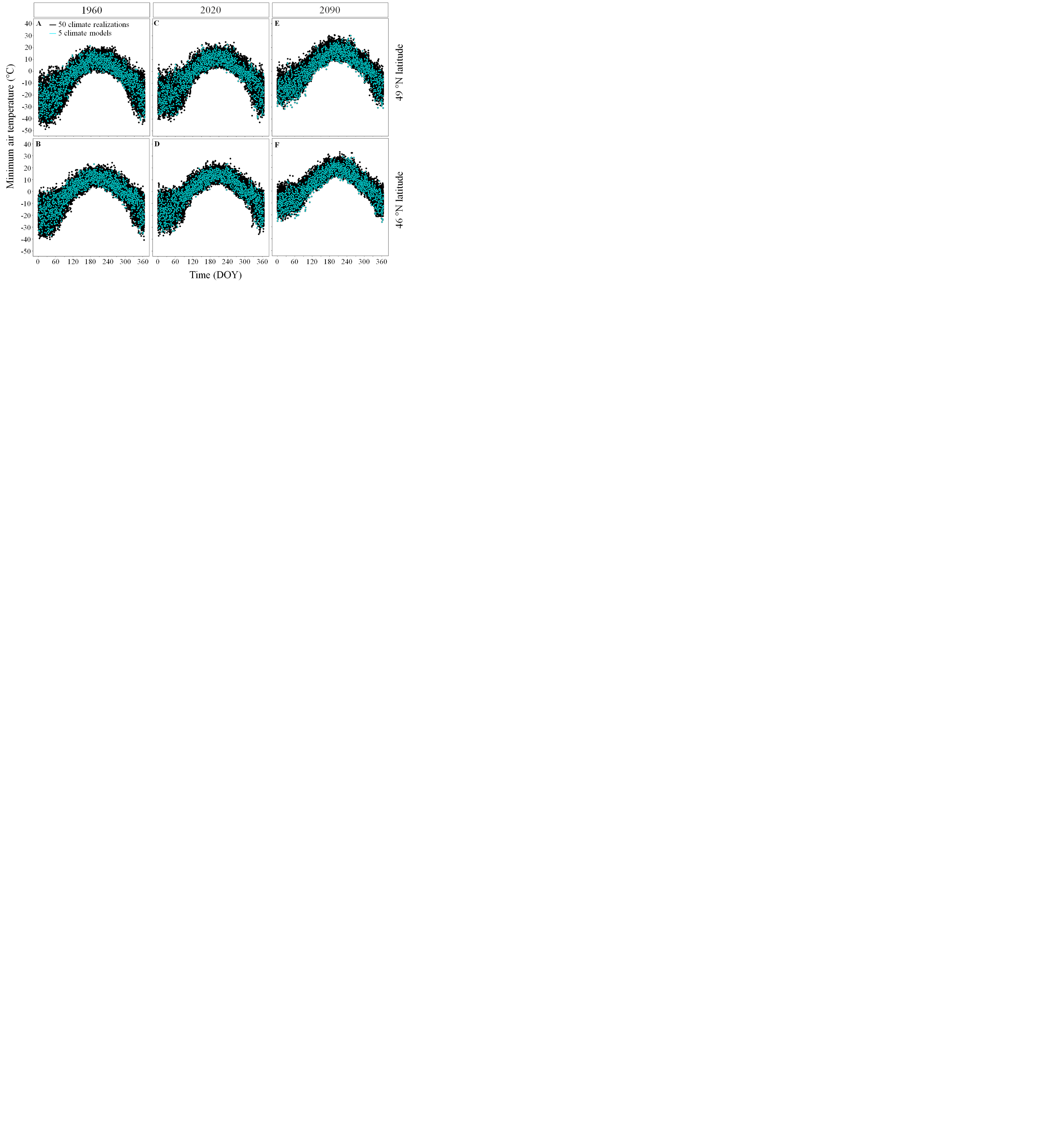


**Fig. S2. Inter- and intra-climate model variability in simulated minimum daily air temperature at two latitudes and for 1960, 2020, 2090.**


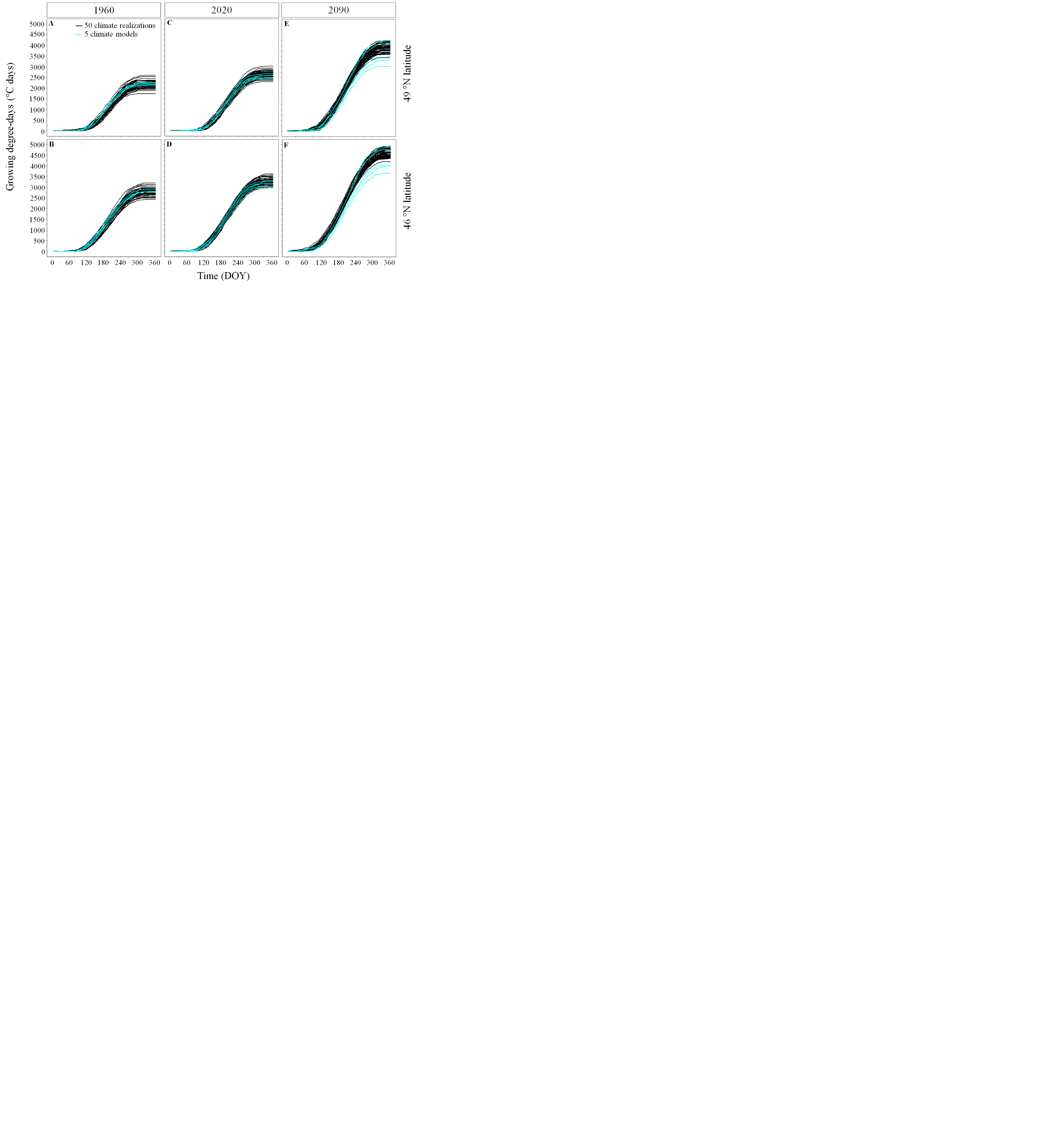


Fig. S3. Inter- and intra-climate model variability in simulated growing degree-days (0 °C threshold) at two latitudes and for 1960, 2020, 2090.

**Importance of chilling requirement**

A study conducted on boreal tree species in Eastern Canada showed that if the chilling hours dropped below 400 it would delay the timing of budbreak and subsequent growth (Man et al., 2017). This study used this set of equation to calculate the chilling hours.

1. 0.159T+0.506 if air temperature is >-3.4 and < 3.5 (T=temperature)
2. -0.159T + 1.621 if air temperature is > 3.5 and < 10.4 (T=temperature)
3. 0 otherwise

Man, R., Lu, P., Dang, Q.-L. (2017). Insufficient chilling effects vary among boreal tree species and chilling duration. Front. Plant Sci., 15, https://doi.org/10.3389/fpls.2017.01354.

However, we used daily data; therefore, we divided the 400 chilling hours by the 24 hours which gives a daily sum value of 16.7 chilling requirement. Then, we calculated the daily chilling sum, based on the above set of equations, starting on DOY 200 (mid July) until DOY 365. If 16.7 chilling units accumulated by DOY 365, the chilling requirement would be met before growing-degree days could start accumulating. Chilling would thus, play a marginal role at predicting the timing of budbreak.

The chilling values for a southern grid cell and a northern grid cell showed that by 2099 and under the warmest RCP scenario (8.5), 16 chilling unit were met by DOY 365 for 99 % of the simulations. Moreover, growing degree-days only start accumulating when the mean air temperature is above 0 ˚C, but chilling units could still accumulate when temperature was between -3.4 ˚C and 0 ˚C. Therefore, chilling units could keep accumulating even after December 31 without delaying the start of GDD accumulation. If some specific species would need more chilling, it is very likely that they would meet their chilling requirements prior growing degree-days could start accumulating.

Out of the 14500 climate simulation analyzed for chilling requirements (50 climate simulations over 145 years at two grid cells = 50 × 145 × 2 = 14500), only 67 did not reach 16 chilling units by DOY 365, out of these 67 simulations, 25 simulations reached 15 chilling units, 20 simulations reach 14 chilling units, 14 simulations reach 13 chilling units, and only 8 simulations were below 13 chilling units. Recall that these simulations could still reach the required chilling since chilling keeps accumulating from Janurary to April, which was not even considered in this analysis. Therefore, 99% of climate simulations that were analyzed reached the 16 chilling unit threshold prior January 1^st^ thus, the timing of budbreak we predicted was not affected by not considering the chilling effect in our analyses.


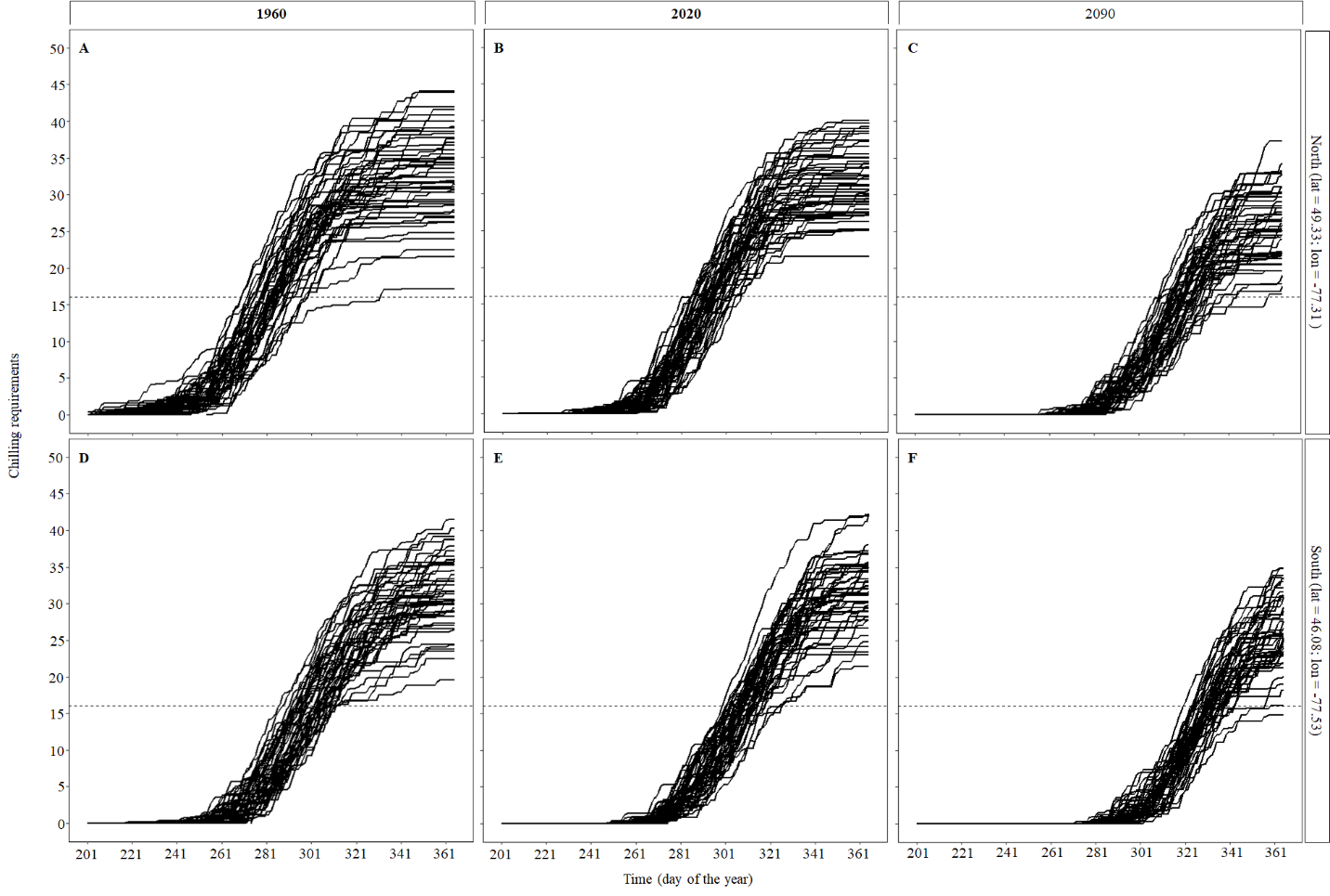


Fig. S4. Intra-model variation in the cumulative sum of the chilling units at two latitudes for 1960, 2020, 2090. The dashed line represents the threshold under which budbreak could be substantially delayed due to lack of chilling.
